# Unraveling the link: locomotor activity exerts a dual role in predicting Achilles tendon healing and boosting regeneration in mice

**DOI:** 10.3389/fvets.2023.1281040

**Published:** 2023-12-21

**Authors:** Melisa Faydaver, Mohammad El Khatib, Valentina Russo, Mara Rigamonti, Marcello Raspa, Oriana Di Giacinto, Paolo Berardinelli, Annunziata Mauro, Ferdinando Scavizzi, Fabrizio Bonaventura, Valentina Mastrorilli, Luca Valbonetti, Barbara Barboni

**Affiliations:** ^1^Unit of Basic and Applied Biosciences, Department of Biosciences, Agro-Food and Environmental Technologies, University of Teramo, Teramo, Italy; ^2^Tecniplast S.p.A., Buguggiate, Italy; ^3^National Research Council, Institute of Biochemistry and Cell Biology (CNR-IBBC/EMMA/Infrafrontier/IMPC), International Campus ‘A. Buzzati-Traverso’, Rome, Italy; ^4^PLAISANT S.r.l., Rome, Italy

**Keywords:** Achilles tendon, locomotor activity, histomorphology, extracellular matrix, home-cage monitoring, tendon regeneration

## Abstract

**Introduction:**

Tendon disorders present significant challenges in the realm of musculoskeletal diseases, affecting locomotor activity and causing pain. Current treatments often fall short of achieving complete functional recovery of the tendon. It is crucial to explore, in preclinical research, the pathways governing the loss of tissue homeostasis and its regeneration. In this context, this study aimed to establish a correlation between the unbiased locomotor activity pattern of CRL:CD1 (ICR) mice exposed to uni- or bilateral Achilles tendon (AT) experimental injuries and the key histomorphometric parameters that influence tissue microarchitecture recovery.

**Methods:**

The study involved the phenotyping of spontaneous and voluntary locomotor activity patterns in male mice using digital ventilated cages (DVC^®^) with access to running wheels either granted or blocked. The mice underwent non-intrusive 24/7 long-term activity monitoring for the entire study period. This period included 7 days of pre-injury habituation followed by 28 days post-injury.

**Results and discussion:**

The results revealed significant variations in activity levels based on the type of tendon injury and access to running wheels. Notably, mice with bilateral lesions and unrestricted wheel access exhibited significantly higher activity after surgery. Extracellular matrix (ECM) remodeling, including COL1 deposition and organization, blood vessel remodeling, and metaplasia, as well as cytological tendon parameters, such as cell alignment and angle deviation were enhanced in surgical (bilateral lesion) and husbandry (free access to wheels) groups. Interestingly, correlation matrix analysis uncovered a strong relationship between locomotion and microarchitecture recovery (cell alignment and angle deviation) during tendon healing. Overall, this study highlights the potential of using mice activity metrics obtained from a home-cage monitoring system to predict tendon microarchitecture recovery at both cellular and ECM levels. This provides a scalable experimental setup to address the challenging topic of tendon regeneration using innovative and animal welfare-compliant strategies.

## 1 Introduction

Tendinopathies account for a significant proportion (~30%) of reported musculoskeletal conditions, with multiple predisposing factors, including overuse, aging, and endocrine/metabolic diseases (i.e., obesity and diabetes), impacting approximately 2–5% of the general population (1–4). Athletes are particularly susceptible, with approximately 10% of runners developing Achilles tendinopathy and tendon injuries, constituting roughly 50% of all sports injuries. Tendinopathy leads to persistent pain, swelling, stiffness, and loss of strength in the affected area (1–4).

Current treatments, including surgical approaches (i.e., autografts, allografts, and xenografts) and pharmacological symptomatic treatments, such as non-steroidal anti-inflammatory drugs, corticosteroids, and cryotherapy (5, 6), have limited success (7). Spontaneous recovery of tissue homeostasis in adult organisms is inefficient due to the poor cellularity, vascularization, and slow metabolism of tendon tissues (7, 8). This often results in partial tissue healing and scar tissue formation (7, 8). In most tendinopathies, the pathology takes a chronic course, increasing the likelihood of recurrences as re-ruptures, pain, impairment of movement, and disability (9, 10).

Tendinopathies that do not lead to tissue rupture or widespread damage involve prolonged rehabilitation protocols, affecting the patient’s quality of life (11). Physical therapy is frequently considered as the sole treatment (11–15). In particular, movement therapy, mainly focused on resistance exercise, is considered the cornerstone of conservative management (16), encouraging load tolerance and leading to structural adaptations at the musculotendinous unit and to functional restoration (17, 18). However, its effectiveness is influenced not only by the specific exercises but also by the magnitude of the stimulus, quantified by the concept of exercise dose (19). On the other hand, immobilization seems to decrease tendon water and proteoglycan content while increasing the amount of collagen cross-links (20, 21). While movement therapy aims to facilitate healing, prevent the development of adhesions, and increase the range of motion (12, 13), the underlying mechanisms are yet to be understood. Overall, the complex correlation between clinical outcomes and histological aspects in tendinopathies remains a topic of investigation.

Perspectives for future innovation in tendon diagnosis and therapy are increasingly focused on gaining a better understanding of the mechanisms driving ECM and tissue microarchitecture remodeling, ultimately leading to biomechanical tissue recovery, which is currently poorly understood (22). In this biomedical challenge, experimental mammals take on multiple functions by covering the role of patients awaiting clinical solutions, working as preclinical models to gather crucial biological information and serving as translational models to drive advancements in medicine. In this latter context, the laboratory mouse (*Mus Musculus*), the most widely used translational model in basic science research and clinical practice, has also been employed to investigate tendon-related mechanisms.

In addition to its potential, limited exploitation of the mouse as preclinical model can be attributed to the small size of the structures, which complicates tissue histological/molecular assessment and biomechanical testing recorded under standardized settings. Existing practices, including tissue harvesting, intra-body devices (23), and surgical methods (24), although successful in observing tendon tissue healing, have limitations related to animal welfare and the sacrifice of multiple animals. This necessitates the development of non-invasive strategies for predicting tissue recovery. Digital ventilated cages (DVC^®^) are advanced animal housing systems that provide controlled environments for laboratory animals (25–27). These non-invasive systems consist of a combination of electronics and software elements to collect data directly from the home cages without disturbing the subjects, thus preserving their well-being (25–27). They also enable the collection of high-resolution spatiotemporal data on activity patterns without attaching external sensors or devices to the animals, thereby preserving their natural and spontaneous behaviors. As of today, there are no published studies on the usage of DVCs for monitoring the movement activity of mice to predict tendon regeneration. The current study aims to test whether the recovery of tissue microarchitecture and ECM deposition correlate with motion data collected through an automated non-invasive technology using a mouse AT model.

Starting from these premises, the present study was designed using a validated surgical mouse AT injury model (28, 29) to identify the existence of a correlation between morphological and morphometric tissue parameters, ECM healing, and functional data collected from animal locomotion activity using an automated setup. This approach could be helpful in developing digital markers capable of predicting tendon recovery.

The study focuses on exploring tendon injuries within the context of quadrupedal locomotion, seeking to illuminate nuanced aspects of functional recovery. The experimental group was formed by comparing quadrupedal locomotion in subjects with unilateral and bilateral surgical AT lesions. The choice between unilateral and bilateral injuries is grounded in the replication of distinctive injury scenarios mirroring the clinical spectrum observed in human and animal patients. Unilateral injuries offer insights into the repercussions of isolated tendon damage, mirroring scenarios commonly encountered in clinical practice where a single limb is affected. In contrast, the inclusion of bilateral injuries aims to simulate more severe and widespread tendon damage, akin to situations where both limbs are compromised. By employing both unilateral and bilateral injury models, this study aims to unravel the intricate interplay between injury severity and functional recovery, enhancing the ecological validity of our findings. Furthermore, to evaluate if homeostatic regulation of tendon microarchitecture could be influenced in response to additional mechanical loading, a group of mice was housed in cages with unrestricted access to running wheels, providing a source of additional voluntary motor activity. It is hypothesized that, regardless of the lesion type, access to running wheels could enhance the functional activity of the mice and accelerate tendon recovery, including microarchitecture and ECM remodeling.

To this end, the morphological and morphometric parameters selected to analyze tendon explants 28 days after surgery aimed to document ECM remodeling and repair by assessing cell count and alignment, COL1 deposition and organization, and vascularity. In addition, post-surgical functional recovery was documented by collecting movement data, with 24/7 access, of the male mouse strain CRL:CD1 (ICR) using the digital ventilated cage (DVC^®^) developed by Tecniplast S.p.A.

The present results demonstrated that non-intrusive surveillance revealed a correlation between some key microarchitectural tendon parameters such as cell alignment, angle dispersion, and unbiased collected night activity data. These results offer, for the first time, the possibility of using a scalable activity monitoring system (DVC^®^) to predict the clinical evolution of tendon healing. This validation of a reproducible non-invasive experimental setup can impact the refinement of preclinical tendon-related settings, including endpoints, to enhance reproducibility and promote a better understanding within the research community.

## 2 Materials and methods

### 2.1 Ethics statement

The animal experimentation conducted at CNR-IBBC (EMMA/Infrafrontier) received authorization from the Italian Ministry of Health under authorization number 183/2021-PR on 12/03/2021. This was carried out in compliance with the guidelines outlined in the Italian Legislative Decree 04/03/2014 n. 26, which implements Directive 2010/63/EU concerning the protection of animals used for scientific purposes.

### 2.2 Home cage activity monitoring: DVC^®^ system

Animal activity was measured by using the DVC^®^ home-cage monitoring system. This technology provides non-intrusive, 24/7 monitoring of individual or group animal activity within their home cage, offering high-resolution data on their activity levels over time (25, 26). The system consists of an electronic sensing board equipped with 12 uniformly distributed contactless electrodes positioned beneath each cage, capable of detecting changes in electrical capacitance resulting from animal movements. In this study, the smoothed animal locomotion index (DVC^®^ Analytics, Tecniplast S.p.A.), which is based on the activation density metric (27), was used to assess mouse activity within the cage. The recorded locomotor activity was analyzed during both day- and night-time, starting from the surgery day and continuing up to 27 days post-injury. Additionally, the locomotor circadian rhythm was evaluated by observing the average activity in 3 h intervals throughout the 24 h day. We also considered the daily average activity within the 12 h light and dark periods. In this study, the conducted analysis focused exclusively on locomotor activity. Ethical and practical considerations constrained the feasibility of conducting biomechanical tests and gait analysis to assess functional data.

### 2.3 Mice

For this study, we selected specific pathogen-free (SPF) CRL:CD1 (ICR) male mice, known for their spontaneous dynamic locomotor activity (25). These mice were housed within the EMMA/Infrafrontier international research infrastructure at the CNR-IBBC Core Structure (Monterotondo, Rome, Italy).

In this study, the choice of male mice aimed to minimize biological variables, facilitate surgical procedures due to their larger size, align with epidemiological data indicating a higher incidence of acute tendinopathy cases in males, and account for the lower tendency for week-to-week variance in activity, as previously observed (26).

Before the surgical procedure, mice were grouped in standard cages, with 3–5 individuals per cage. Following the surgery, the animals were individually housed in IVC-DVC^®^ racks for the entire study duration, with *ad libitum* access to a standard diet (4RF21; Mucedola). The mice were kept under controlled environmental conditions (i.e., temperature = 21 ± 2°C; relative humidity = 55 ± 15%). They were maintained on a 12-h light/12-h dark cycle (7 AM–7 PM, lights on) with 50–75 air changes per hour (ACH) and a 12:12 light cycle. The light intensity at the room level was 230 lx, with slight variations based on cage position within the rack, ranging from 29 to 12 lx. The cages were equipped with certified dust-free wood bedding (Scobis one; Mucedola). The mice had access to chlorinated and filtered water *ad libitum*, and no cage changes were adopted from the surgery day until the end of the experiment (28 days post-surgery).

It is important to highlight that, due to technological constraints with the DVCs and the necessity to capture individual activity levels, the animals were housed solitarily. The potential impact on animal welfare associated with individual housing is recognized, and a precautionary measure of conducting daily observations throughout the experiment was implemented. These observations were aimed at monitoring for signs of anxiety or behavioral issues and, reassuringly, no such indications were observed.

The cages were equipped with a solitary running wheel (either blocked or free) and paper nesting material with bedding for environmental enrichment. This form of enrichment can be effectively used in DVCs without affecting the detection of animal movement, which is based on the comparison of consecutive capacitance measurements and is not influenced by the presence of static objects. Food supplementation was administered 14 days after the surgical procedures.

### 2.4 Experimental plan

**Figure 1:**
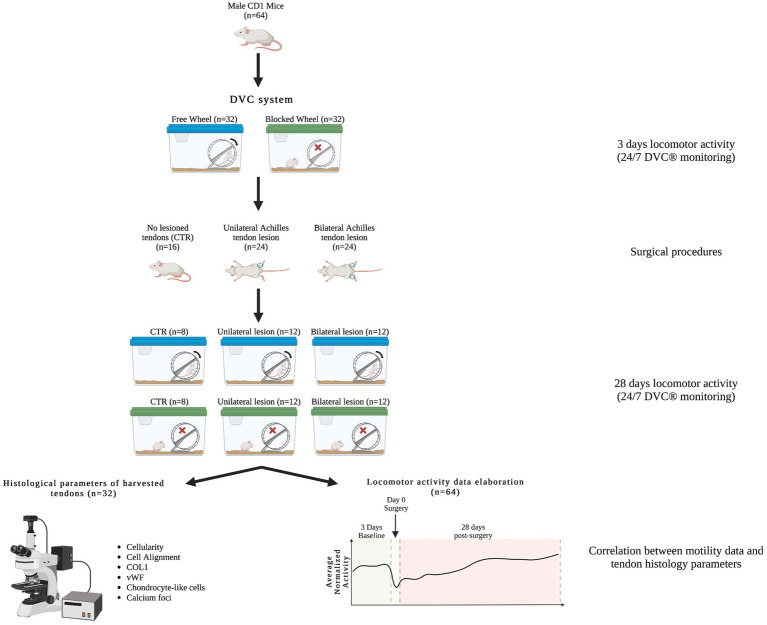
Animal experimental design. Workflow of the animal experimental design, in which different male CRL:CD1 (ICR) mice were randomly placed in DVC^®^ systems with free and blocked running wheels for locomotor activity monitoring (24/7). The CRL:CD1 (ICR) mice were subjected to two types of Achilles tendon injury, unilateral and bilateral, while mice with non-lesioned tendons were used as controls. In the Figure, a representative scheme shows the assessment of histomorphometric parameters and motility data elaboration for their correlation.

A total of 64 male CRL:CD1 (ICR) wild-type adult mice (*Mus Musculus*) aged 10–12 weeks old were first randomly divided into two different tissue injured models: uni- and bilateral AT lesions. These two groups of impaired animals were further analyzed under two housing conditions: cages equipped with free running wheels to provide additional movement opportunities and cages equipped with blocked wheels. A group of single housed control (CTR) mice (non-lesioned tendon), with both free and blocked running wheels, were added. The preclinical experimental plan is documented in Figure 1 and Table 1.

**Table 1:** Number of subjects used for this study.

	Automated locomotion activity monitoring			Histology and morphometric analyses		
	CTR mice	Mice with unilateral lesions	Mice with bilateral lesions	CTR tendons	Unilateral lesioned tendons	Bilateral lesioned tendons
Blocked wheel	8	12	12	8	6	12
Free wheel	8	12	12	8	6	12
Total mice	64			32		

All surgical procedures were performed under stringent aseptic procedures, general anesthesia, and analgesia. Analgesia was also extended for post-operative pain management. Specifically, anesthesia was induced with a 4% isoflurane oxygen mixture (Abbott Laboratories, Maidenhead, UK) at 4 L/min and maintained with a 1.5–2% isoflurane/oxygen mixture at 2 L/min. Anesthetized animals were placed on a heating pad in the sternal position and the hindlimbs were extended caudally. All procedures were performed under a stereomicroscope. After trichotomy and skin disinfection, an incision of approximately 6/7 mm was performed along the posterior aspect of the leg, between the musculotendinous junction of the gastrocnemius muscle and the calcaneus. When the skin was separated, the AT was identified using microsurgical forceps, and was exposed and isolated from the nearby superficial flexor tendon (SFT or plantar tendon) of the fingers. A lesion with a loss of substance less than 50% of the total diameter of the AT was created in the center of the length of the tendon (3 mm), using a scalpel blade. The creation of the tendon lesion was performed either unilaterally or bilaterally depending on the experimental plan (Figure 1). Mice, after post-anesthesia recovery, were immediately ambulated to promote regeneration. Throughout the operation, the tendon was kept moist and well-hydrated with sterile saline solution. The surgical incision was sutured with 6–0 monofilament. An analgesic (Buprenorphine 0.05 mg/kg) and an antibiotic (Enrofloxacin 0.2 mg/kg) were administered to prevent post-operative pain or septic complications, although SPF health category animals are pathogen-free and are thus less susceptible to postoperative infections. During the entire study period (from 1 week before tendon injury to 28 days post-induced lesion), the mice were housed in an IVC-DVC rack developed by Tecniplast S.p.A. at the CNR-IBBC and EMMA/Infrafrontier facilities to collect animal activity data directly from the home cage. The DVC system allows automatic measurement of animal activity 24/7 (26). Specifically, the measured activity levels in this study pertained to the DVC plate and did not encompass wheel usage. This limitation arose from the absence of sensors on the traditional wheels attached to the cages, hindering the recording of mouse movement on the wheels. Despite this constraint, this gap was addressed by conducting daily observations, carried out by an operator who provided valuable insights into the mice’s engagement with the running free wheels.

Animals were euthanized by cervical dislocation 28 days post-surgery, according to the literature (30–35). After euthanasia, sampling was carried out by harvesting the tendons and preparing them for histological analysis. The histological assessment was performed 28 days post-injury, when spontaneously healed tendons within the early-term recovery are expected to still undergo scar repair, characterized by disorganized ECM and infiltration of somatic and immune cells, as reported previously (30–35).

### 2.5 Histology detection of tissue microarchitecture recovery

Morphometric investigations were performed *ex vivo* on the samples detailed in Table 1. Briefly, AT samples, from which myotendinous and osteotendinous junctions were removed, were fixed in 4% paraformaldehyde/phosphate-buffered saline (PBS) for 1 h. They were then dehydrated using a series of alcoholic solutions ranging from 70 to 100% and embedded in paraffin wax. For histological analysis, samples were sliced to a thickness of 7 μm using a microtome and placed on poly-lysine adhesion microscope slides. The paraffin was removed through immersion in xylene for 10 min, followed by a series of alcohol solutions (from 100 to 70%) for 30 s and distilled water to rehydrate the sample.

The following stainings were used for histological analyses:

- Alcian Blue: polysaccharide staining, especially GAGs, as an indirect staining for chondrocyte cells.
- Alizarin Red: to detect the presence of calcium deposits in a tissue.

### 2.6 Immunofluorescence assessment of tendon ECM remodeling

Immunohistochemical (IHC) studies were conducted using previously validated antibodies for mice (Table 2) on samples fixed following the above-mentioned protocol. After washing with PBS, non-specific binding was blocked by incubating the sections at room temperature (RT) in PBS/1% bovine serum albumin (BSA) for 1 h. Tissue sections were incubated with the primary antibody overnight at RT, and subsequently exposed to the secondary antibody for 1 h at RT. The nuclei were stained with DAPI (Sigma-Aldrich, St. Louis, MO, USA, D9542 1:100). The following IHC stainings were performed:

- COL1: to assess the fiber organization.
- Von Willebrand factor (vWF): to precisely target and stain the vWF present in blood vessels, enabling the assessment of angiogenesis and the presence of blood vessels inside the injured tendon.

**Table 2:** Details of primary and secondary antibodies used for IHC.

Primary antibody	Dilution	Secondary antibody	Dilution
COL1 (Novus Biologicals, Centennial, USA, Code: NB600-408)	1:600	Alexa Fluor 488 anti-rabbit (Abcam, Cambridge, UK, Code: AB 150077)	1:200
vWF (DakoCytomation, Denmark, Code: A 0082)	1:400		

Specific antibodies used for these analyses are detailed in Table 2.

### 2.7 Morphometric analysis

**Figure 2:**
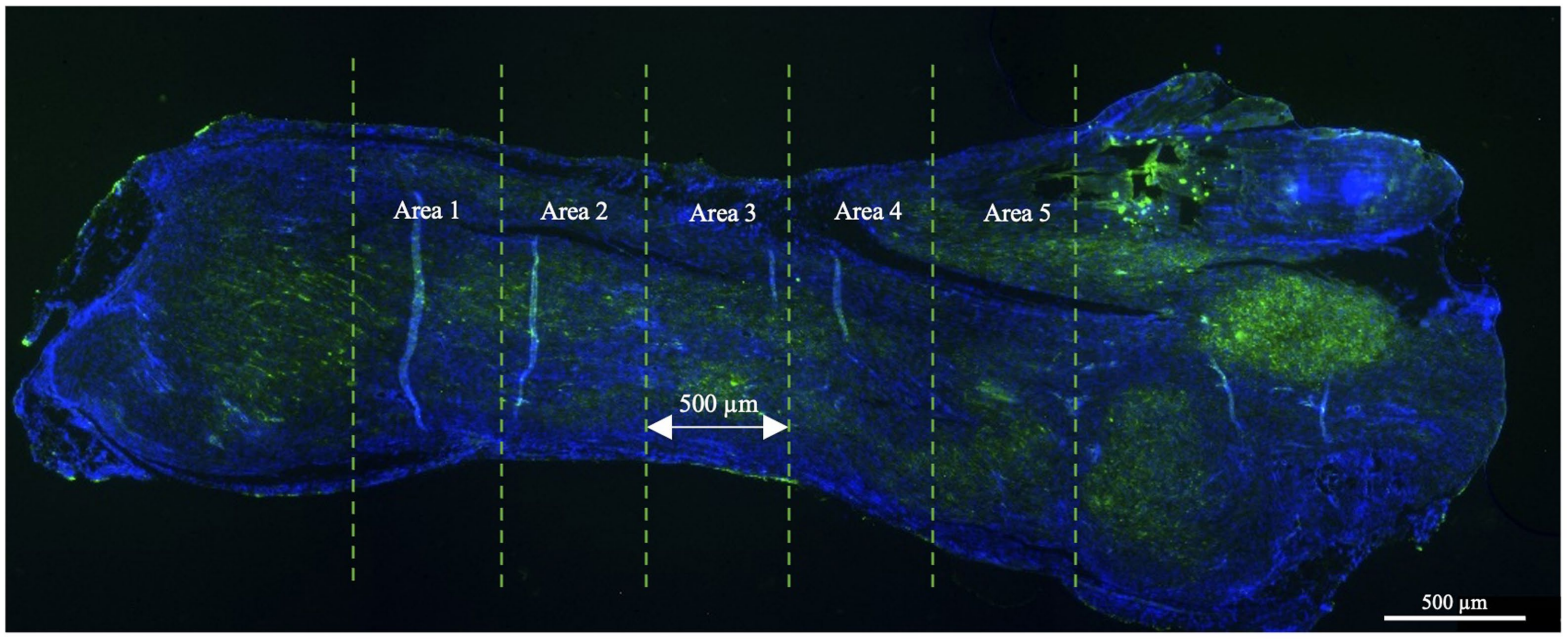
The large image format of an injured tendon tissue as observed under a fluorescence microscope. For the morphometric analyses, the tendon was divided into five equal areas of 500 μm each and analyzed according to the parameters explained below. The lesion core was represented by area 3 while areas 1 and 2 and areas 3 and 4 belonged to the areas located toward the myotendinous junction and the enthesis, respectively. Only tendon tissues were harvested, and the sections related to tendon extremities (myotendinous junction and the enthesis) were excluded from the analysis.

Morphometric analyses were conducted using an Axioscop 2+ epifluorescence microscope (Zeiss, Oberkochen, Germany) equipped with a cooled color charge-coupled device camera (CCD; AxiovisionCam, Zeiss) and an interactive and automatic image analyzer (Axiovision, Zeiss). All assessments were conducted at ×40 magnification by acquiring, through a person blinded to experimental conditions, four randomly selected fields from each of the five connecting areas throughout the tendon, arranged from top to bottom. Areas 1 and 2 represented the areas located toward the myotendinous junction while areas 4 and 5 were located toward the enthesis area. Area 3, situated at the core of the lesion, was chosen for analysis. The total analyzed area size was 1.85 mm^2^ (Figure 2). Morphometric analyses were performed using both quantitative and semi-quantitative methods.

Quantitative analyses were conducted on the images of the histological and immunohistochemical (IHC) stainings using guided programs to measure the following parameters:

- *Cellularity*: quantified by counting the number of DAPI-stained nuclei in each field using the Cell Profiler software built-in tool “IdentifyPrimaryObjects.” One of the main features of Cell Profiler is its ability to perform quantitative analysis of biological images, including cell counting, which has been used in several other applications (36–39).
- *Cell Alignment and Angle dispersion*: cell dispersion refers to the spreading out or scattering of cells within a tissue. This parameter was assessed using the directionality plugin in ImageJ software. The elaborated data are expressed as direction and dispersion, which reflect the tallest peak observed at the center of the Gaussian and the Gaussian’s standard deviation (S.D.). An increase in dispersion values indicated that cell orientation was not homogeneous. Following that, the direction values were standardized to the control (CTR) samples of healthy AT.

Semi-quantitative analyses were conducted to assess COL1 fiber organization, vascularity, chondrocyte infiltration, and calcification, as outlined in Table 3.

**Table 3:** Scoring system used for quantitative and semi-quantitative histomorphometric parameters.

Parameter	Score 0	Score 1	Score 2	Score 3
Cellularity expressed as the average of cell N° in CTR and treated samples within all analyzed areas/average of cell N° in healthy tendons	Fold change >3	3 < fold change >2	2 < fold change <1.3	Fold change <1.3
Cell alignment and angle dispersion expressed as the ratio between the angle orientation of tested groups *vs.* healthy tendons along the longitudinal axis of the tendon	Ratio > 3 corresponding to an irregular cell distribution and alignment along the longitudinal axis of the tendon	3 < ratio > 2.1 corresponding to cells that start to acquire a parallel orientation to the longitudinal axis of the tendon with high variability	2 < ratio > 1 or ratio < 1 corresponding to cells that start to acquire a parallel orientation to the longitudinal axis of the tendon with a dispersion higher than 25%	Ratio < 1 corresponding to cells that acquire a parallel orientation along the longitudinal axis of the tendon with a dispersion <25%
COL1 fiber alignment	No COL1 expression and no organized fibers within the injured tendon	COL1 expression with organized fiber formation corresponding to max. two areas within the injured tendon	COL1 expression with scattered aligned fibers noted in max. four areas within the injured tendon	COL1 expression with aligned fibers noted in all five areas within the injured tendon
Vascularity	Vascular plexus	Presence of blood vessels that are either scattered or aligned to the longitudinal axis of the tendon for an extension corresponding to max. one area within the injured tendon	Blood vessels aligned to the longitudinal axis of the tendon for an extension corresponding to one up to four areas within the injured tendon	Few blood vessels oriented along the longitudinal axis of injured tendons (as healthy tendons)
Chondrometaplasia	Chondrocyte foci are present in all five areas	Chondrocyte foci are present in up to four areas throughout the tendon	Chondrocyte foci are present in up to one area throughout the tendon	No presence of chondrocytes
Osteometaplasia	Calcification foci are present in all five areas	Calcification foci are present in up to four areas throughout the tendon	Calcification foci are present in up to one area throughout the tendon	No calcification foci

A score of 0 represents the lowest healing level while a score of 3 represents the highest healing level and is the closest to a healthy tendon tissue.

A scoring system, summarized in Table 3, was adopted according to a previously validated and published system (32), with minor modifications in order to assess quantitative and semiquantitative morphometric data to define tendon healing profiles. The scoring analyses were independently conducted by two blinded assessors.

To assess the inter-assessor reliability in attributing scores to the semi-qualitative parameters, three key metrics were calculated. The percent agreement (%) reflects the proportion of cases where assessors assigned the same scores. Cohen’s kappa (κ) measures the level of agreement beyond chance, accounting for the possibility of agreement occurring by random chance. Weighted kappa (κ_w_) provides a similar assessment, but with a consideration for the degree of disagreement, offering insights into the strength of agreement for weighted categorical data (40–42). These metrics are crucial for understanding the consistency in scoring across diverse parameters. The results of these assessments are presented in Supplementary Table 1.

### 2.8 Statistical analyses

The average absolute and normalized daily day- and night-time activity over time (from day 0 up to day 27 post-surgery) was assessed by using Gaussian linear mixed models, with the lmerTest R software package used to model data and test for fixed effects (Type III ANOVA with Satterthwaite’s method). We considered the intercept as a random effect, allowing it to vary by cage. We used a top-down approach and successive likelihood ratio tests to define the model and the variables best explaining the data. The average activity within 3-h bins over a 24 h day was assessed by using the rank-based analysis of variance-type statistic (ATS), as implemented in the nparLD R software package (43). The factors were considered statistically significant if *p* < 0.05.

Quantitative and semi-quantitative data from the *in vivo* experiments were assessed for their normality distribution by D’Agostino Pearson. The data were expressed as mean ± S.D., and one-way ANOVA was employed, followed by a Tukey *post hoc* test, to compare all tested animals belonging to healthy tendons (used as control), injured tendons with unilateral or bilateral lesions, and those with blocked or free wheel access. Statistical analysis for assessing activity normalized by the CTR group was performed using a two-tailed independent t-test. GraphPad Prism 9 (GraphPad Software, San Diego, CA, USA) was used for data analysis. The values were considered statistically significant for at least *p* < 0.05. Correlations between movement activity and quantitative histological data were performed on the registered and calculated measurements for each animal. Confidence intervals for Pearson’s correlation coefficients were calculated using a Fisher’s Z’ transformation (GraphPad Prism 9, GraphPad Software, San Diego, CA, USA). Data were statistically significant where *p* < 0.05.

## 3 Results

### 3.1 DVC 24/7 activity in tendon injured mice

**Figure 3:**
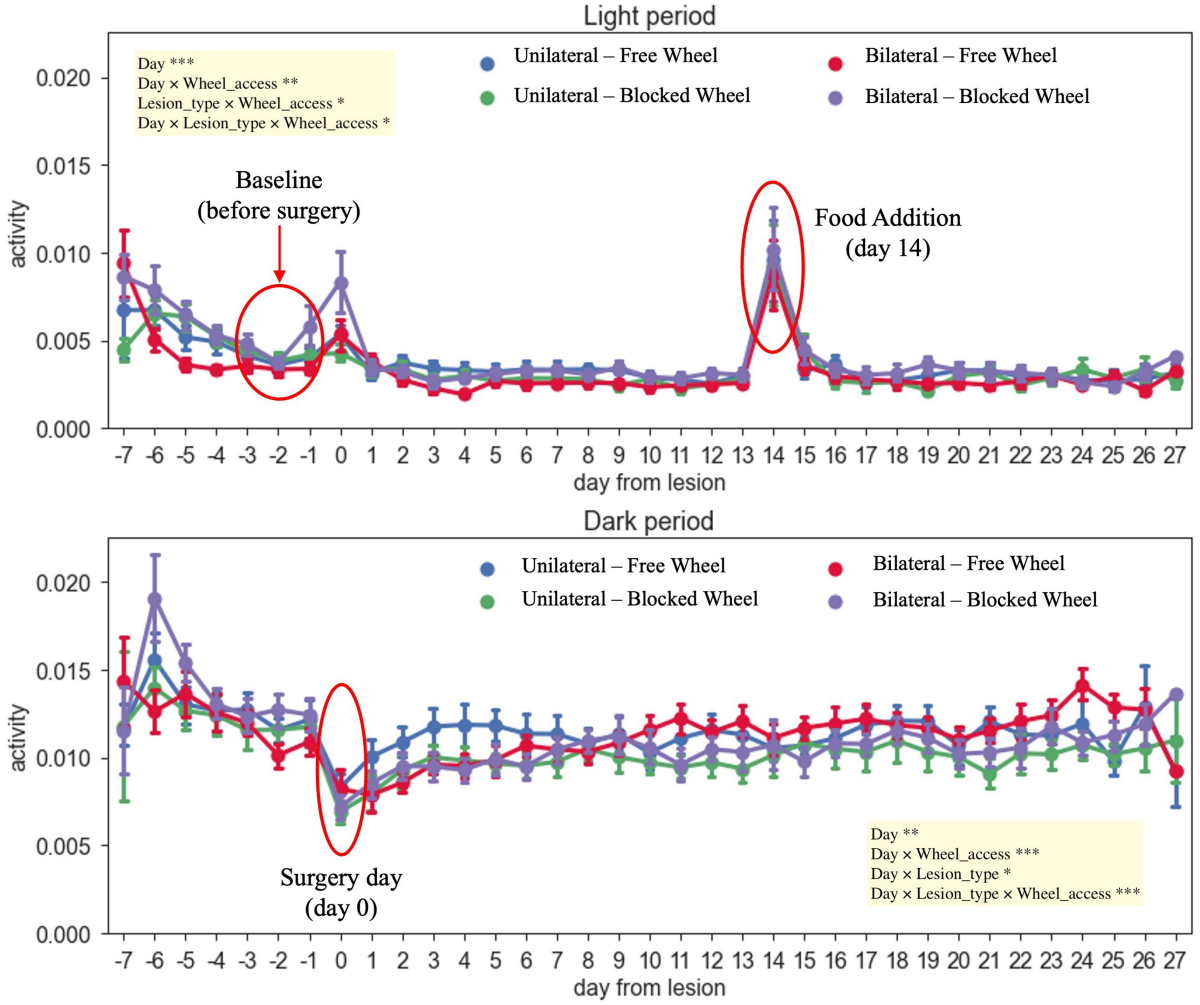
Daily activity during light/dark hours throughout the recovery period of the mice. The different colors of the graph represent the different analyzed groups (free and blocked wheel groups and unilateral and bilateral groups). The light period was from 07: 00 AM until 07: 00 PM while the dark period was from 07: 00 PM until 07:00 AM. The data are presented as the mean ± SEM, and significant fixed effects are indicated in each panel (*p < 0.05, **p < 0.01, and ***p < 0.001).

The DVC system continuously monitored the activity of mice both during the day and night for a total of 34 days, including 7 days prior to the surgeries and 28 days post-surgeries (Figure 3). Of note, the measured activity levels were specifically related to the mice’s activity on the DVC plate and did not take into consideration their use of the running wheel. As expected, mice exposed to a light/dark cycle of 12/12 h are generally more active during the dark period (26). The average daily light activity displayed two notable peaks corresponding to surgery (day 0) and to addition of food (day 14 post-injury); thus, these days were excluded from the analysis. During the night-time, instead, all animals reached their minimum amount of activity on the day of injury (day 0), as shown in Figure 3.

No significant main differences between unilateral and bilateral groups were found in day-time activity from day 0 (surgery day) up to 27 days post-injury (main effect of lesion-type, *F*_1,69_ = 0.1211, *p* > 0.05). During the night-time, all injured animals, independently from the type of lesion and wheel access, started to recover and again became quite active immediately after the surgery day (day 1), with notably faster recovery within unilaterally lesioned mice with free wheel access, up to 7 days post-injury (Figure 3). Moreover, the activity continued to slightly increase throughout the healing period up to 27 days for all groups (main effect of day, *F*_1,1512_ = 8.6024, *p* < 0.01), with a higher increase registered within bilaterally lesioned animals with free wheel access (interaction effect between day, lesion type and wheel access, *F*_1,1512_ = 12.6506, *p* < 0.001).

**Figure 4:**
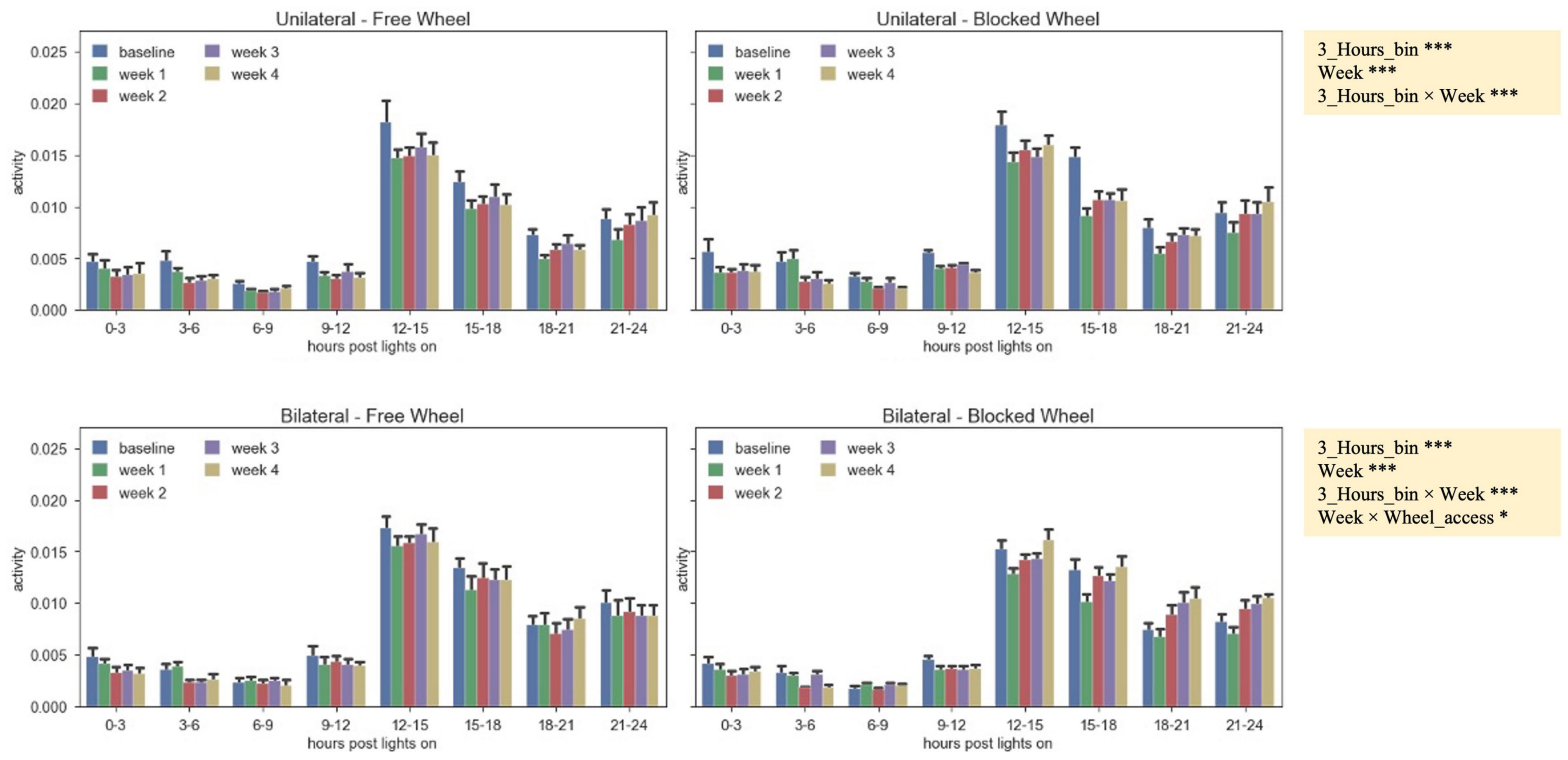
Activity pattern during the 24 h of the day (lights-on + lights-off), represented by the average absolute activity during 24 h, split into 3-h bins for unilateral and bilateral groups, with free and blocked access to the wheel, and aggregated by the baseline period and the 4 weeks after surgery. The data are presented as the mean ± SEM, and significant fixed effects are indicated in each panel (***p < 0.001) and for bilateral lesions (*p < 0.05 and ***p < 0.001).

The distribution of the daily average activity was divided into eight bins of 3 h each, starting from the time of lights-on (Figure 4). These patterns were observed during the baseline period and the 4 weeks following surgery. It was observed that all animals, independent of their groups, exhibited reduced activity during the first 12 h after lights-on, with the activity peaking in the 12–15-h bin just after lights-off. Subsequently, the activity gradually decreased until the 21–24-h bin. By comparing both unilaterally and bilaterally lesioned groups, it emerged that all animals displayed significantly lower activity in the first 2 weeks when compared to the baseline, with a gradual increase over time (nparLD test, period factor; unilateral: statistic = 10.3658, DF = 2.9786, *p* < 0.0001; bilateral: statistic = 8.9343, DF = 3.0186, *p* < 0.0001; Figure 4). There were notable differences in activity levels between the 12–15- and 15–18-h bins after lights-off (nparLD test, interaction between period and 3-h bin; unilateral: statistic = 3.4926, DF = 10.2410, *p* < 0.001; bilateral: statistic = 6.5196, DF = 8.9209, *p* < 0.0001; Figure 4). However, the activity distribution over the 24 h did not significantly differ based on wheel access, neither in mice with unilateral nor bilateral lesions (nparLD test, interaction between wheel access and 3H bin; unilateral: statistic = 0.5885, DF = 3.2055, *p* > 0.05; bilateral: statistic = 2.1282, DF = 3.0023, *p* > 0.05; Figure 4).

### 3.2 Effects of husbandry and experimental procedures on mouse night activity

**Figure 5:**
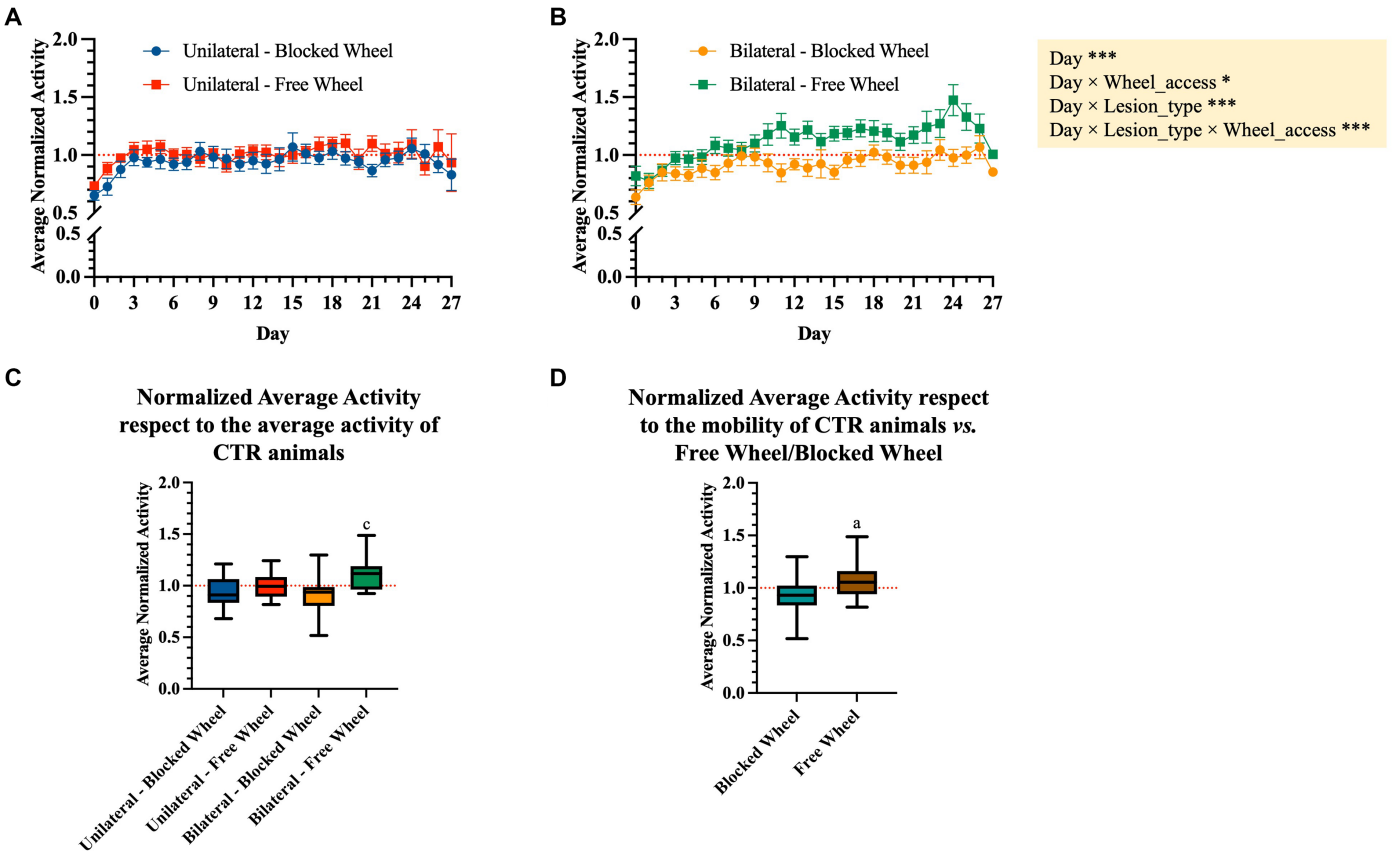
Normalized average activity during the dark period. Daily night-time average activity between day 0 and 27 post-surgery was normalized to the average night-time activity of the last 3 days before surgery in WT mice subjected to **(A)** unilateral and **(B)** bilateral lesions. The data are presented as the mean ± SEM, and significant fixed effects are indicated in each panel (*p < 0.05 and ***p < 0.001). **(C)** Box plots representing the average movement activity normalized to the baseline (3 days pre-surgery) and to the corresponding control group. Statistically significant values were set up for *p* < 0.05. ^c^ *vs.* bilateral-blocked wheel. **(D)** Aggregated normalized average activity *vs.* wheel access independent of the lesion type. Statistically significant values were set up for *p* < 0.05. ^a^ *vs.* blocked wheel. The quantitative data are expressed as the mean with maximum and minimum values.

To analyze the evolution of activity after surgery regardless of the amount of activity, the average daily night recorded activity was normalized by dividing it by the average night activity of the last 3 days before surgery, used as a baseline reference period. As shown in Figures 5A,B, unilaterally and bilaterally lesioned mice initially showed activity levels lower than the baseline up to 3 days post-surgery, with significant differences over time between unilateral and bilateral lesion mice groups (interaction effect between day and lesion type, *F*_1,1241_ = 27.4361, *p* < 0.0001; Figures 5A,B). Interestingly, the average normalized activity showed a different trend over time depending on the wheel type (interaction effect between day and wheel access, *F*_1,1241_ = 6.0790, *p* < 0.05; Figures 5A,B), especially within mice subjected to bilateral lesions that exhibited a significantly higher increase of activity when accessing the free wheel compared to the blocked wheel (interaction effect between day, lesion type, and wheel access, *F*_1,1241_ = 12.0340, *p* < 0.001; Figures 5A,B).

The nocturnal normalized activity of each tested group was then aggregated and normalized to their corresponding CTR (blocked and free wheel access, Figures 5C,D). In detail, the obtained aggregated data showed no significant differences between the unilaterally lesioned mice undergoing spontaneous healing, indifferent to wheel access type (Figure 5C). On the contrary, bilaterally lesioned mice with free wheel access showed a significantly higher normalized average activity with respect to those with blocked wheel access (*p* < 0.05, Figure 5C). Interestingly, the normalized average activity was significantly dependent on the wheel access type, with significantly higher values within the free access wheel (*p* < 0.01, Figure 5D).

### 3.3 Tendon microarchitecture and ECM remodeling are both affected by injury type (uni- *vs.* bilateral lesions) and husbandry conditions (cages with free *vs.* blocked wheels)

The main morphological parameters defining the process of tendon healing have been analyzed to assess how injury lesions (unilateral *vs.* bilateral) and husbandry conditions (homing in cages with free *vs.* blocked wheels) may impact structural tendon healing, before moving toward the statistical correlation between morphometric tissue data and locomotion activity.

#### 3.3.1 Cellularity

**Figure 6:**
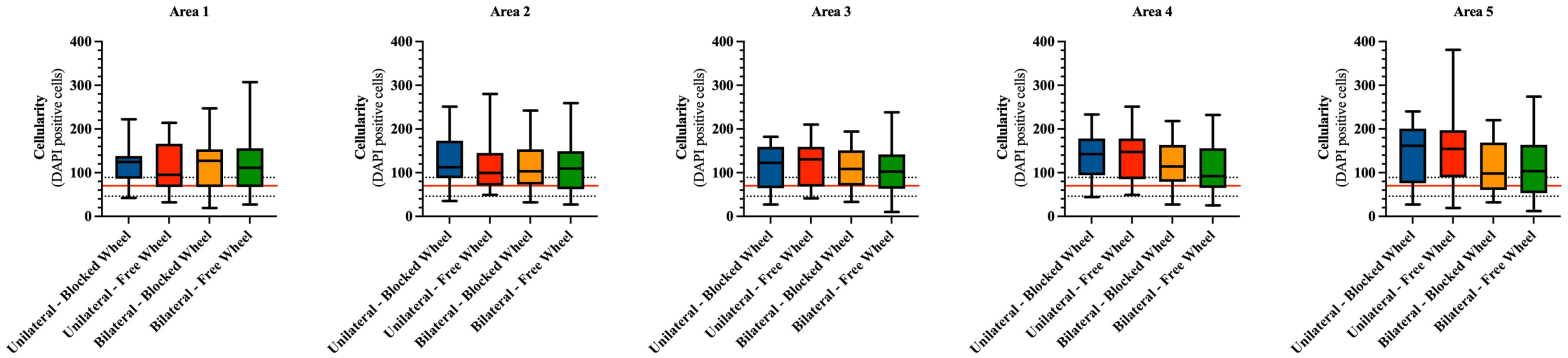
Cellularity spatial distribution in mice Achilles tendons 28 days post-injury. The number of cells is calculated from a total of five different areas of four different fields each and represented as box plots showing the mean with maximum and minimum cell values within each analyzed group. The cellularity of healthy tendons is represented by the horizontal red line (mean) and the corresponding black dotted lines (maximum and minimum). The quantitative data are expressed as the mean with maximum and minimum values.

**Figure 7:**
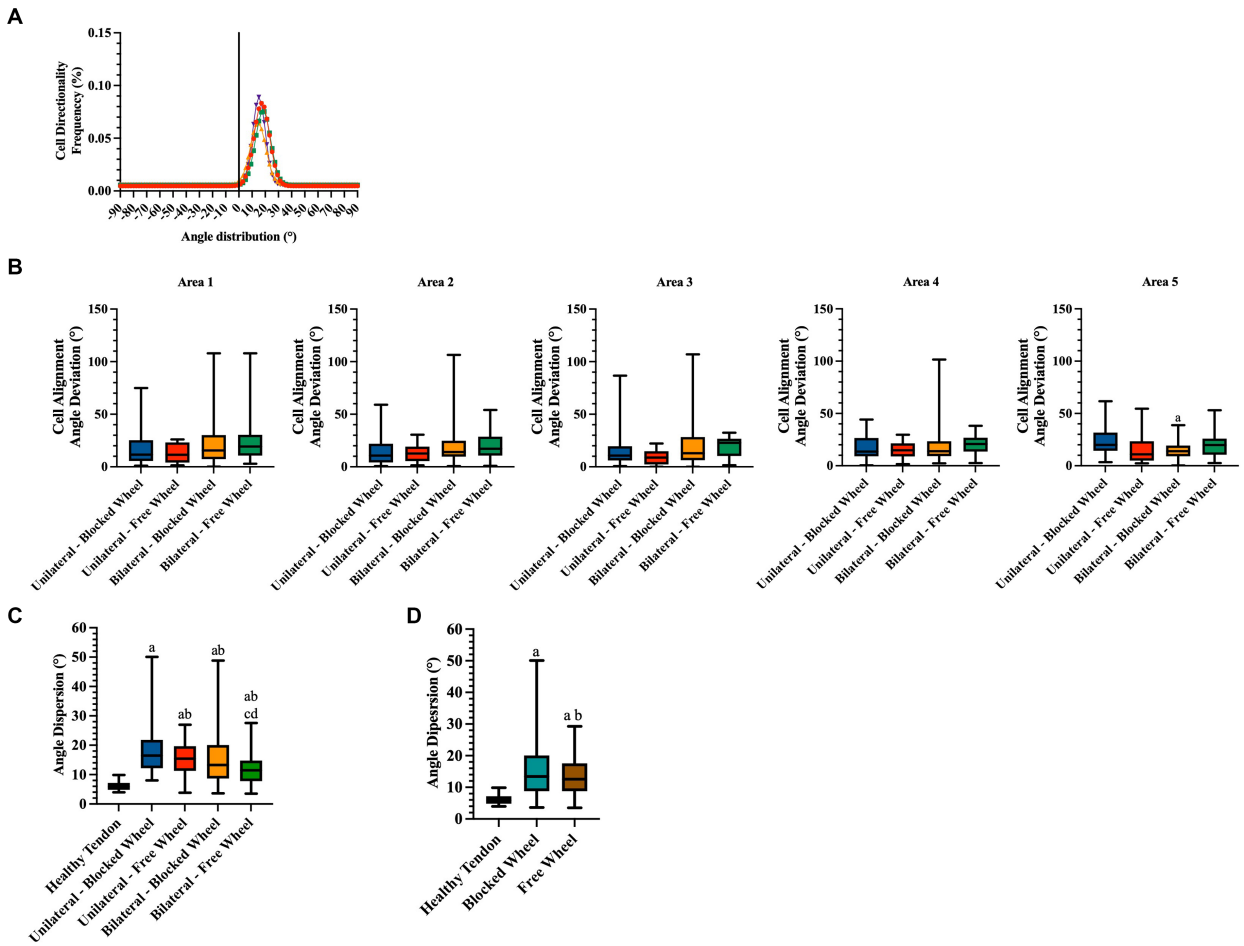
Directionality analyses on cell orientation of the different groups at 28 days of spontaneous healing. **(A)** Representative directionality curve of the healthy tendons assessed through directionality of ImageJ. **(B)** Box plots showing the angle deviation within spontaneously healing tendons subjected to different conditions within all analyzed areas, normalized to the healthy tendon area used as a reference for the analyses at 28 days. Statistically significant values were set up for *p* < 0.05. ^a^ statistically significant *vs.* unilateral-blocked wheel. The quantitative data are expressed as the mean with maximum and minimum values. Box plots representing the angle dispersion (the standard deviation of the Gaussian) **(C)** amongst the different tested groups. Statistically significant values were set for *p* < 0.05, with the indicated superscripts ^a^ *vs.* healthy tendon, ^b^ *vs.* unilateral-blocked wheel, ^c^ *vs.* unilateral-free wheel, and ^d^ *vs.* bilateral-blocked wheel. **(D)** Comparison of the influence of wheel access type independent of lesion type. A healthy tendon was used as a reference sample for the analyses. Statistically significant values were set for *p* < 0.05 ^a^ *vs.* healthy tendon and ^b^ *vs.* blocked wheel group.

To evaluate the potential impact of lesion type and wheel access on tendon microarchitecture tissue recovery, cellularity (Figure 6) and cell alignment (Figure 7) analyses were performed. A spatial approach was adopted, dividing the lesioned tendons into 5 areas of 500 μm each, with area 3 located in the surgical injury zone and areas 1 and 5 positioned near the myotendinous junction and the enthesis, respectively (for detailed information, refer to materials and methods section).

Initially, statistical analyses were carried out to compare cellularity between healthy tendons (CTR) and experimental tissues on an area-by-area basis. Hypocellularity was a prevalent feature in CTR tissues, with an average of approximately 70 cells per unit area (indicated by the red line in Figure 6).

In detail, hypercellularity was observed in areas 1, 2, 3, and 4 in all experimental groups (*vs.* CTR: *p* < 0.05) with the exception of unilateral lesioned free access within area 1, where the average cellularity was lower and comparable to that of healthy tissues (unilateral lesioned free access *vs.* CTR; *p* > 0.05). Hypercellularity persisted near the enthesis zone (area 5), exclusively in unilateral lesioned tendons, regardless of the wheel access group (both unilateral lesioned free and blocked access *vs.* CTR; *p* < 0.01), while no significant differences were observed within the bilateral lesioned groups (for both *vs.* CTR; *p* > 0.05).

On the other hand, the statistical analyses conducted to identify differences among the areas within the experimental or husbandry groups did not reveal any significant variations (Figure 6).

#### 3.3.2 Cell alignment

Two additional morphological quantitative parameters highly indicative of tendon regeneration, namely, cell alignment and nuclei orientation (Figure 7), were evaluated. Angle deviation was determined by calculating the difference between the cell alignment directions of the lesioned groups in comparison to the cell alignment observed in the CTR group (Figure 7B).

Healthy tendon cell alignment exhibited a sharp Gaussian curve with an average angle distribution of 17.02° ± 1.86 (Figure 7A). The angle deviations did not exhibit any significant differences among the experimental groups in all areas (Figure 7B). An exception was noted in the angle deviation in area 5 of bilateral lesioned animals housed in cages with blocked wheel access, which was significantly lower than the unilateral group (bilateral-blocked wheel *vs.* unilateral-blocked wheel; *p* < 0.05).

To gain a deeper understanding of the impact of lesion type and husbandry on tendon healing, the angle dispersions (standard deviation of the Gaussian curves) were analyzed. According to Figure 7C, healing tendons showed significantly higher angle dispersions than healthy tendons (all experimental groups *vs.* CTR; *p* < 0.05). However, the values of angle dispersion were notably influenced by tendon lesion type. In particular, bilateral injured tissues displayed significantly lower angle dispersion values (bilateral *vs.* unilateral; *p* < 0.05) with the lowest angle dispersion recorded in bilateral tendon lesions housed in a cage with free wheel access (experimental lesioned groups *vs.* bilateral-free wheel; *p* < 0.05).

Statistical analysis of the cell angle dispersion values was also conducted to gain insight into the effect of husbandry conditions (Figure 7C). In this context, significantly lower angle dispersion values were recorded in mice housed in cages with free wheels, highlighting the role of additional motion in promoting tendon recovery.

#### 3.3.3 COL1 deposition

**Figure 8:**
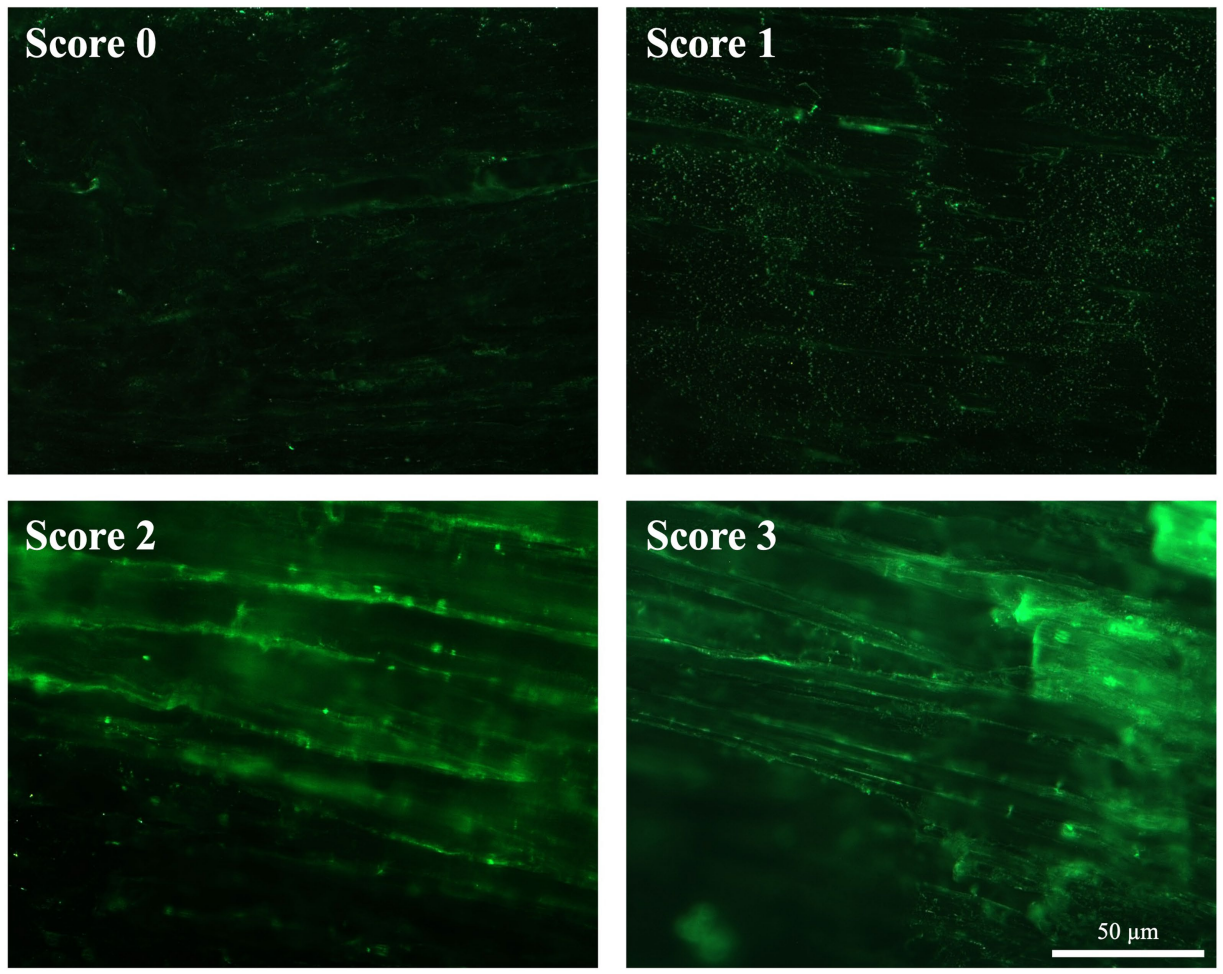
ECM organization in terms of COL1 after spontaneous healing in injured tendons. Representative images of each attributed score for COL1 organization (green fluorescence) 28 days post-injury.

COL1 deposition in AT was assessed with IHC (Figure 8). In order to classify the progression of tendon healing, a qualitatively scoring system (Table 3) ranging from 0 (no COL1 deposition) to 3 (COL1 deposition and organization similar to CTR healthy tendon) has been adopted.

In detail, CTR displayed a highly organized COL1 ECM characterized by parallel fibers showing a homogeneous fluorescence intensity throughout the whole tissue (score 3; Figure 8). On the contrary, the experimental injured tendons showed different degrees of disorganized ECM, characterized by a non-homogenous COL1 fluorescence distribution (Figure 8). In detail, IF revealed that mice belonging to bilateral lesion groups housed in cages with free wheels showed a more regular COL1 deposition, with several areas displaying aligned fibers (up to 4). This latter experimental group achieved the highest average score (1.64 ± 0.73), whereas the scores of the other experimental groups ranged from 0.83 ± 0.44 (unilateral-free wheel) to 1.17 ± 1.03 (unilateral-blocked wheel) with regularly deposited COL 1 fibers that never reached three areas (Figure 8).

Of note, the COL1 deposition scores attributed by the two assessors were reliable according to the calculated percent agreement of 87.87%, Cohen’s κ score of 0.827, and κ_w_ of 0.881 (Supplementary Table 1).

#### 3.3.4 Blood vessel remodeling

**Figure 9:**
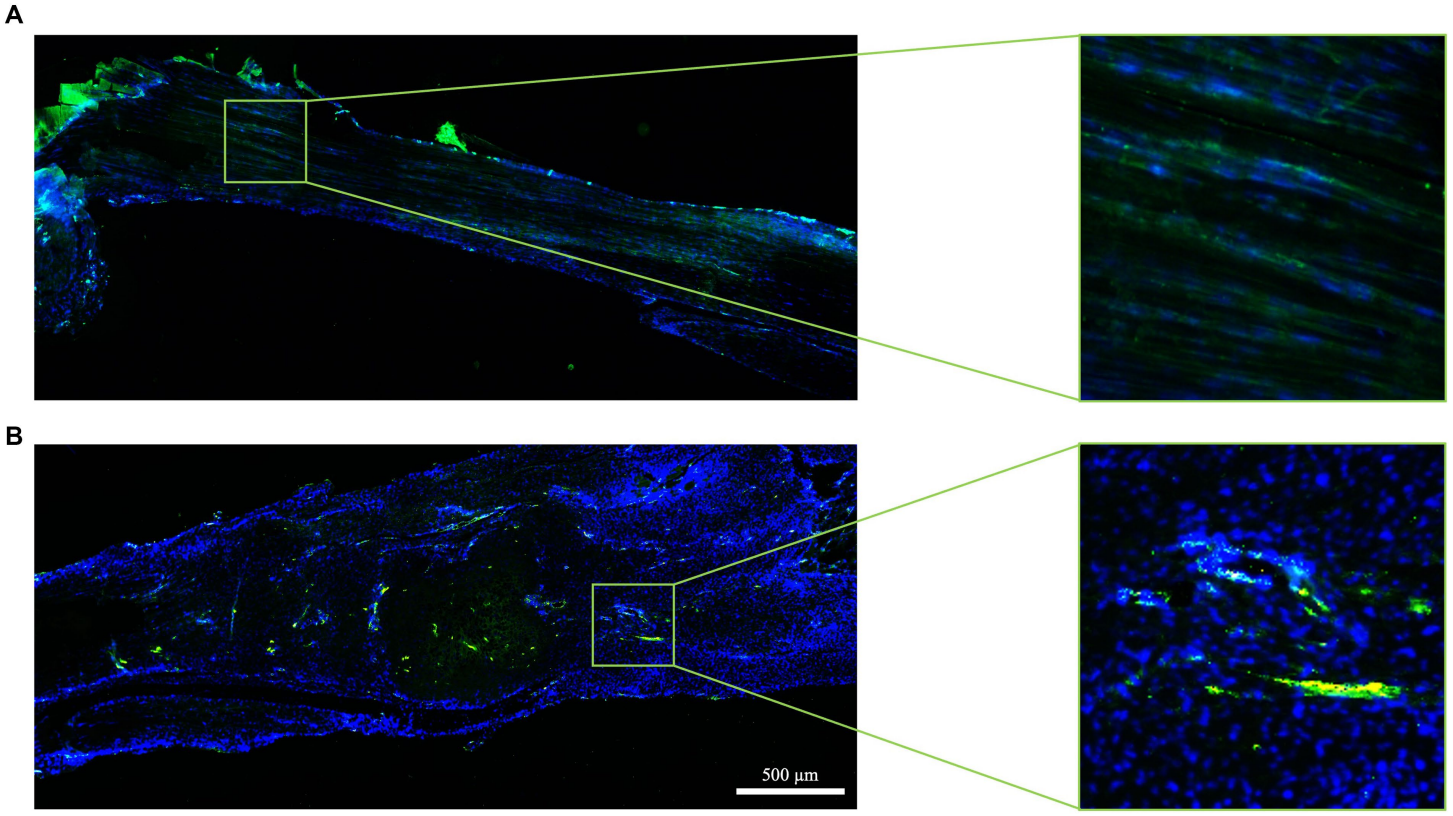
Tendon vascularization within healthy and spontaneously healed tendons. Blood vessel distribution is documented in **(A)** healthy (score 3) and **(B)** spontaneously healed tendons (score 0) at day 28 post-injury by immunohistochemistry using the endothelial marker vWF (green fluorescence). Nuclei were counterstained with DAPI. In each panel, five areas are shown, with area 3 representing the lesion core.

Blood vessel remodeling has also been associated with tendon healing. As depicted in Figure 9, a few blood vessels, aligned along the longitudinal axis of the tendon, were predominantly situated in CTR, mainly around the paratenon and within the endotenon. These vessels exhibited an orientation parallel to the COL1 fibers, resulting in a qualitative score of 3, as per the adopted scoring system (Table 3).

The experimental injury activated the blood vessel remodeling process, primarily in a lesion type dependent manner (Figure 9). Mice with bilateral lesions exposed to free wheel access displayed more advanced organization of blood vessels, with aligned structures observed in four out of six animals, covering most areas (five out of five). In contrast, the qualitative analysis of the blood vessel network deteriorated in unilaterally lesioned tendons, where predominantly scattered blood vessels were observed (Figure 9).

The differences among the experimental groups became more apparent when analyzing the mean score values. Indeed, the histological blood vessel scores in both bilateral lesioned groups were approximately 1.5 times higher (2.33 ± 0.71 *vs.* 1.91 ± 1.38 in the bilateral-free and blocked wheel group, respectively) than those recorded in unilaterally lesioned tissues (1.17 ± 1.33 *vs.* 1.50 + 1.23 in the unilateral-free and blocked wheel groups, respectively).

The scores assigned by the two assessors for vascularity demonstrated reliability, with a calculated percent agreement of 81.25%, Cohen’s κ score of 0.728, and weighted κ (κ_w_) of 0.846 (Supplementary Table 1).

#### 3.3.5 Chondrocytes and calcification foci sites

The combination of Alcian blue and Alizarin red staining was employed to assess cell heterogeneity within the lesioned tendons and to score the presence of chondrogenic ectopic foci or calcification ectopic foci, respectively.

**Figure 10:**
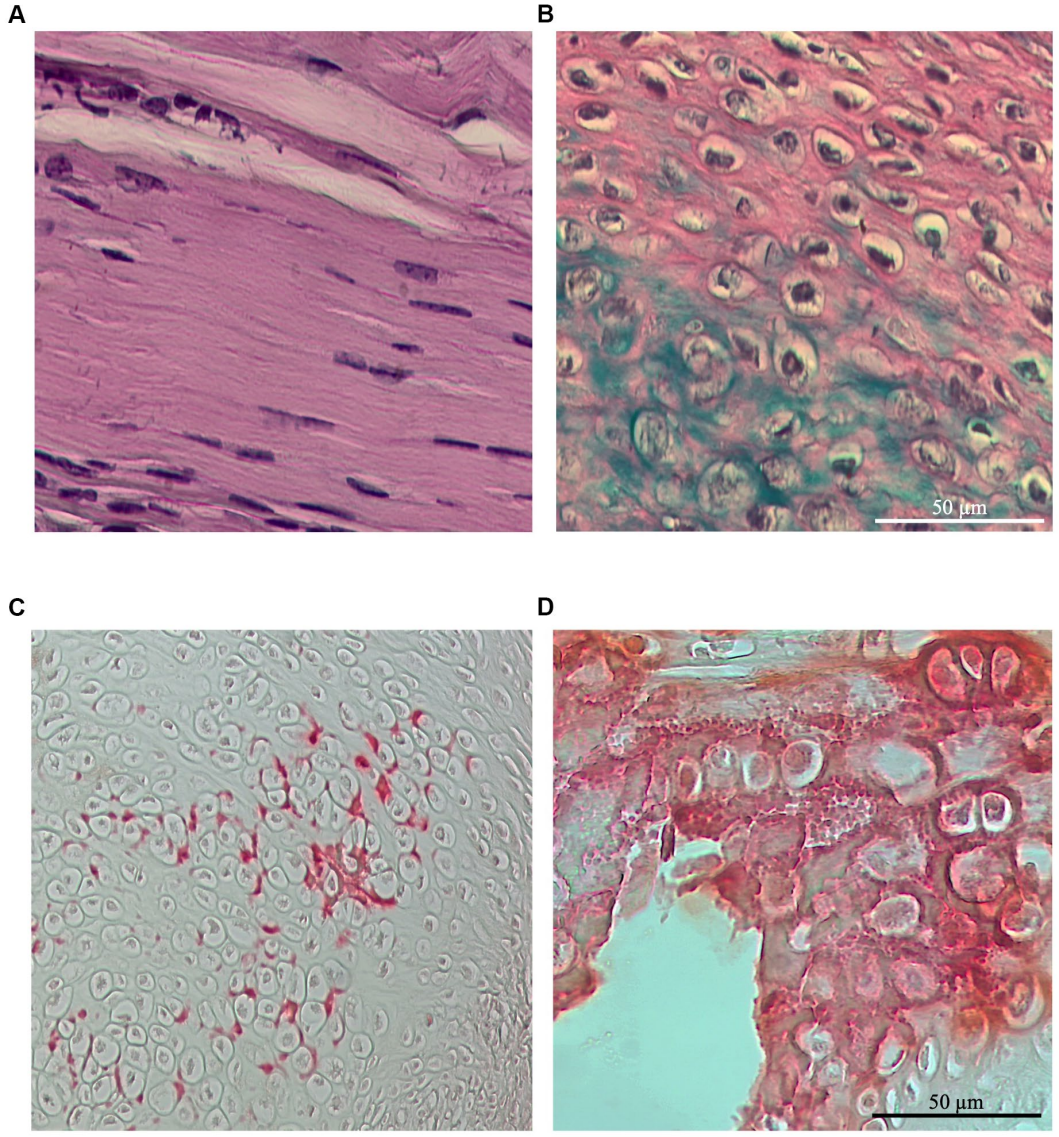
Representative images of Alcian blue staining at 28 days post-injury, revealing chondrocyte infiltration within spontaneously healed tendons depending on the lesion and wheel access types **(A)** located in between the ECM fibers or **(B)** forming aggregates at the site of injury. Representative images of Alizarin red staining at 28 days post-injury, revealing the presence of calcification foci in some of the spontaneously healed tendons, independent of the lesion and wheel access types and localized mainly around the chondrocyte foci **(C)** as preliminary calcium deposits or **(D)** as calcification clusters.

Chondrogenic foci were identified in the majority of regenerated ECM, regardless of the lesion type or husbandry conditions considered. Chondrocytes were primarily observed as rounded stacked cells within the ECM (Figure 10A) and occasionally in aggregate form (Figure 10B). Notably, the chondrogenic scores in all the experimental groups did not exceed 0.22, as observed in the bilateral-free wheel group (*p* > 0.05). The unilateral-free wheel and bilateral-blocked wheel groups both had a chondrogenic score of 0, while the unilateral-blocked wheel group reached a score of 0.17 (*p* > 0.05).

Furthermore, the majority of tendon samples did not exhibit calcification foci, regardless of the lesion type or husbandry conditions considered. Calcification foci were primarily localized around the chondrocytes as initial calcium deposits (Figure 10C) and occasionally as calcification clusters (Figure 10D), occurring in only one area throughout the tendons. Specifically, the calcification scores in all the experimental groups exceed 2.5.

The scores assigned by the two assessors concerning the chondrometaplasia and the osteometaplasia parameters resulted in substantial agreement, with a high percent agreement (93.94 and 84.85%, respectively), κ score (0.786 and 0.680, respectively), and weighted κ (0.804 and 0.716, respectively) (Supplementary Table 1).

### 3.4 Total histological score confirmed that lesion and husbandry conditions influence the process of early tendon healing

**Figure 11:**
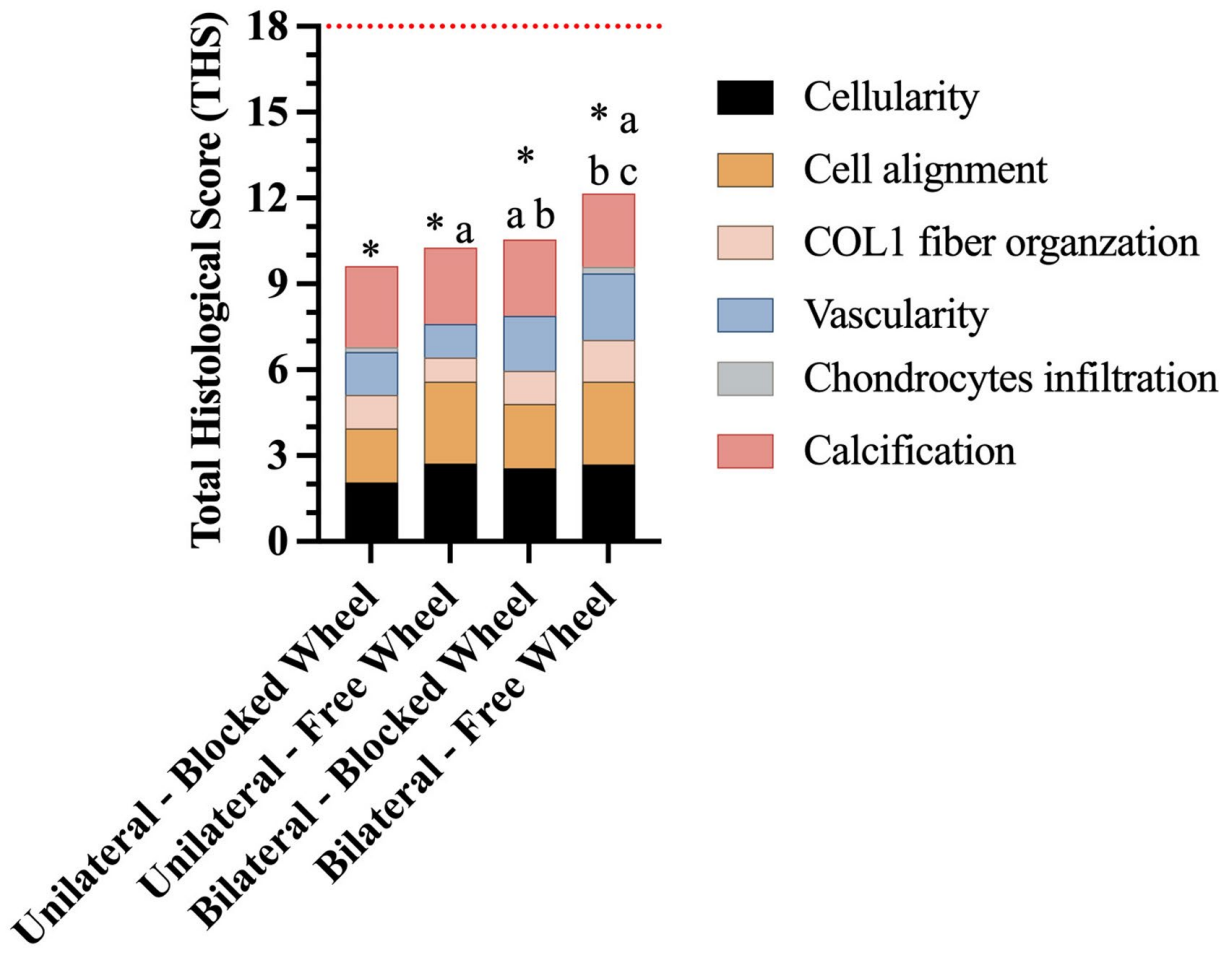
Representative stacked histograms of the semi-quantitative and quantitative histomorphometric score for spontaneously healed tendons at 28 days. The total histological score (THS), represented by its mean, was compared to the healthy tendon (red line), and calculated in terms of cellularity, cell alignment, COL1 fiber organization, vascularity, chondrocyte infiltration, and calcification. Statistically significant values were set up for * *vs.* healthy tendon (*p* < 0.0001), ^a^ *vs.* unilateral-blocked wheel (*p* < 0.05), ^b^ *vs.* unilateral-free wheel (*p* < 0.05), and ^c^ *vs.* bilateral-blocked wheel (*p* < 0.05).

The total histological scores (THSs) achieved by the four experimental groups (Figure 11) were significantly lower than those of healthy tendons (all experimental groups *vs.* CTR; *p* < 0.05; red line in Figure 11). However, the highest THS (12.15 ± 0.18) was achieved in mice with bilateral lesions housed in cages with free wheel access (bilateral-free wheel *vs.* all other experimental groups; *p* < 0.05), whereas the lowest was recorded in the unilaterally lesioned group housed in cages with blocked wheel (9.63 ± 0.19, unilateral-blocked wheel *vs.* all other lesioned groups, *p* < 0.05).

### 3.5 Movement activity is strictly related to the progression of histomorphometric tendon parameters

**Figure 12:**
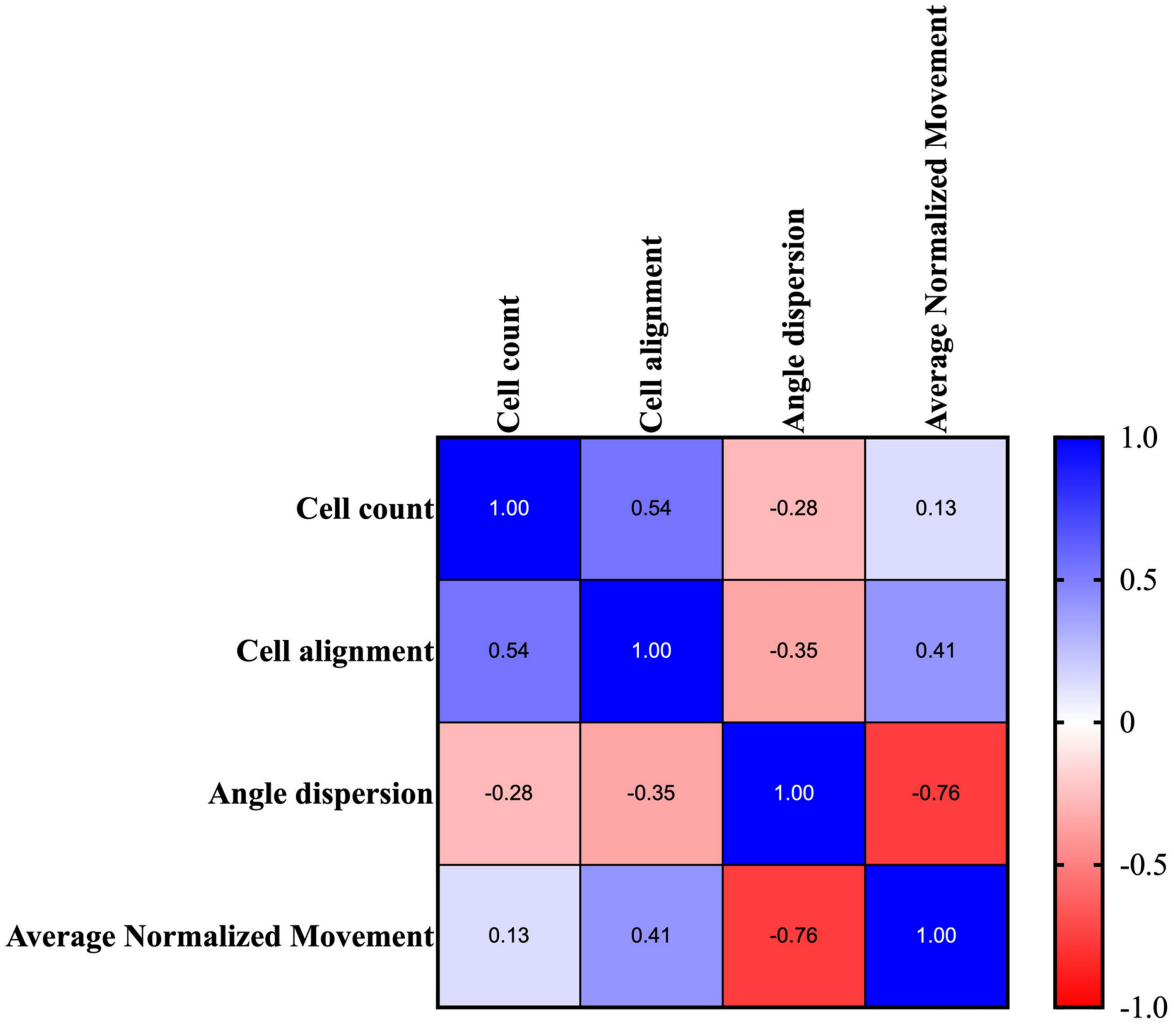
Representative tabular formats of the correlation matrix analysis were performed amongst all quantitative morphological variables. Correlational relationship representation between each quantitative analyzed metric: quantitative histomorphometric measurements *vs.* average normalized movement of the analyzed tendons. Colormap shows the Pearson coefficient factor distribution between 1 and −1 for positive and negative correlation, respectively.

To better understand the existence of a correlation between locomotion and microarchitecture recovery during tendon healing, a statistical Pearson’s matrix analysis was performed. In detail, the average locomotion activity registered by DVC systems during the night period was analyzed in comparison with the quantitative morphometric data (Figure 12).

Of note, the tabular format, summarized in Figure 12, highlighted that cell alignment (Pearson’s coefficient of 0.41; *p* < 0.05) and angle dispersion (Pearson’s coefficient of −0.76; *p* < 0.0001) are strictly correlated with locomotion activity, thus becoming predictive of mouse structural tendon regeneration.

On the contrary, cellularity was positively correlated with cell alignment (Person’s coefficient of 0.54; *p* < 0.01) and did not show any link with the movement data (Figure 12).

## 4 Discussion

Addressing tendon disorders, including those affecting the AT, which accounts for approximately 20% of all cases, remains a significant orthopedic concern. Unfortunately, the tendon healing process often leads to the formation of suboptimal scar tissue, resulting in impaired functionality. Notably, epidemiological studies have demonstrated a rising incidence of tendon ruptures in both athletic and occupational populations. In cohorts from the European Union and Canada (44–46), the incidence of AT ruptures ranges from 6 to 37 per 100,000 inhabitants. This overall increase in incidence can be attributed to degenerative changes observed in the aging population (46), as well as the persistent inferior elastic properties of the healed AT, even in the long term following a rupture (47).

Consequently, this widespread disease has generated an increasing socioeconomic burden, globally estimated at €180 billion. In addition, the absence of predictive diagnostic protocols and effective therapies, combined with increasing life expectancy, has led to a forecast of +25% over the next 5 years. In veterinary medicine, approximately 50% of tendinopathies affect racehorses, resulting in a worldwide loss of €400 billion due to compromised sports performances (48). Given these factors, there is a high clinical demand for addressing tendinopathies in both human and veterinary medicine. Therefore, it is crucial to explore innovative approaches that consider the influence of movement activity and its potential to promote ECM remodeling, with the aim of effectively restoring the normal structure and function of injured tendons.

The present article documents, for the first time, a digital biomarker that predicts tendon microarchitecture regeneration in CRL:CD1 (ICR) mice. Specifically, the implementation of a non-invasive automated home-cage monitoring technology, capable of continuous 24/7 surveillance over weeks or months, plays a pivotal role in this endeavor. Moreover, incorporating automated control systems to regulate environmental parameters, such as temperature, humidity, water supply, and food levels, enhances experimental reproducibility in preclinical settings. Furthermore, the obtained results demonstrated that recovery of locomotor activity is positively correlated with key histological parameters, shedding light on the processes of cellular and ECM remodeling, which are crucial for early tendon healing in experimentally injured AT.

Single housed mice were monitored 24/7 in DVCs, showing both similarities and differences in their spontaneous locomotor activity. As nocturnal animals, all mice were less active during daylight hours, with peak activity occurring during the dark phase, particularly immediately after lights-out, and no significant differences were observed between groups in the distribution of activity throughout the day. Mice subjected to either uni- or bilateral lesions had the lowest spontaneous locomotor activity during both daylight and nighttime hours on the day of injury. Mice began to recover their activities immediately from day 1 post-injury, with the process being faster in mice with access to free wheels compared to those with blocked access. Interestingly, mice subjected to bilateral tendon lesions with free wheel access displayed a greater increase in their spontaneous nighttime activity over time, with higher activity levels at the end of the experiment compared to their baseline period.

All injured animals displayed a regular circadian rhythm, indicating that the experimental injury did not affect their vital functions. Additionally, their recovery was enhanced by voluntary movements provided by the running wheel. Tendon recovery can vary depending on several factors, including injury type and severity, as well as treatment and rehabilitation approaches. Animals with unrestricted access to running wheels showed better activity recovery, contributing to improved early healing performance in bilaterally injured animals compared to unilaterally injured ones. It can be hypothesized that unilateral injury allows quadrupeds to adjust their movement patterns and assume a tripod posture, thereby offering functional protection and partially sparing the injured limb. This adaptation allows the animal to redistribute the load and reduce strain on the injured limb, potentially facilitating the healing process. In contrast, animals with bilateral injuries may not be able to achieve compensatory actions effectively. Despite this, they show the greatest functional recovery, raising questions about the underlying mechanisms. One possibility is that animals with bilateral injuries experience greater biomechanical stress, imposing greater stress on both the injured and non-injured limbs due to the increased demand placed on the remaining limbs during movement and weight-bearing activities, which may trigger adaptive responses contributing to improved functional recovery. These results align with findings that showed improved muscle functional recovery in the case of bilateral volumetric muscle loss injury compared to unilateral (49).

Functional recovery was histomorphometrically verified 28 days post-injury at cellular and ECM levels. To this end, harvested tendons were evaluated for cellularity, cell alignment, COL1 deposition, and vascularity, which were found to be lesion type and wheel access dependent. In particular, tendons from lesioned mice subjected to free running wheels exhibited cell alignment and angle deviation values similar to those of healthy tendons compared to mice with no additional movement (blocked wheels). The most favorable morphometric outcomes were observed in mice with bilateral lesions, with similar pattern observed in COL1 deposition and blood vessel remodeling. Once again, mice with bilateral lesions and unrestricted wheel access displayed ECM homogeneity, with aligned COL1 deposition and organized blood vessels. The obtained data were verified by calculating the overall THS, which showed that the highest values within tendons were derived from mice subjected to bilateral lesions and free wheel access. The overall results reinforced the evidence of the role of lesion type and wheel access in accelerating tendon healing, converging toward the hypothesis of the more targeted regenerative action of bilateral lesions with unrestricted running wheel access. In a study where an end-to-end repair was performed in a simultaneous bilateral AT rupture, the patient experienced a return to full activity after 1 year due to accelerated rehabilitation beginning at 2 weeks for the left limb and 6 weeks for the right, post-operation (50).

Finally, employing correlation matrix analysis revealed a strong correlation between the registered locomotion movement data and two essential morphometric parameters: cell alignment and angle dispersion. It is hypothesized that mechanical stimuli during tendon healing promote better cell re-organization and re-alignment, leading to improved ECM deposition. According to a previously published work, cell alignment and angle deviation have been identified as crucial factors in guiding tendon regeneration and promoting the deposition of organized tendon ECM (32). Mechanical signals, modulated by factors such as SCX, an early marker of tendon development, and TGF, may enhance the transmission of mechanical signals in mice with bilateral tendon injuries and unrestricted wheel activity. This modulation may lead to spatial changes in the actin cytoskeleton of stretched tenocytes, promoting cell alignment and rearrangement and facilitating ECM deposition within the tendon (51–53). Moreover, SCX plays a crucial role in initiating tendon differentiation, while MKX, a crucial transcription factor for tendon development, is involved in tissue growth and collagen fibril assembly (54). Allowing tendon-injured mice to engage in unrestricted movement using a running wheel enables the exploitation of tendon mechanosensitivity and demonstrates the influence of mechanical stimulation on ECM remodeling (55, 56). This process ultimately facilitates the restoration of the injured tendon’s normal structure and function. Within this study, the application of a non-invasive device for monitoring movement pattern activity using the DVCs demonstrated, for the first time, the correlation between movement and microarchitecture tissue recovery, offering a new predictive insight into tendon healing. This validated approach gains further support when delving into the molecular mechanisms involved in tendon regeneration, as specific molecules (genes, proteins, and miRNA) have not been identified yet as specific markers.

Mice appear to recapitulate their functional performances and consequently their histomorphometric features after tendon injury. However, alongside the improvement in regenerating ECM in tendons, a phenomenon of cartilaginous metaplasia emerges, characterized by the presence of chondrogenic foci in nearly all analyzed tendons, irrespective of the lesion type and husbandry conditions. Chondrocytes, typically residing in cartilage, infiltrate the tendon tissue in response to injury or pathological conditions (57, 58), likely migrating from the calcaneal pole of the tendon and undergoing some degree of cell differentiation. This process leads to the deposition of a calcium rich matrix throughout the tendon tissue (57, 58). The incidence of chondrocyte foci formation during tendon healing has been reported in various studies (57–61), underscoring its significance in the healing process.

While the exact prevalence of chondrocyte foci varies depending on the specific tendon and the nature of the injury, it has been observed in a significant proportion of tendon healing cases. De Mos et al. confirmed, using an *in vitro* model, that chondrogenic differentiation is present in midportion Achilles tendinopathy. Moreover, Howell et al. demonstrated that injured tendon healing in adult mice is characterized by fibrovascular scarring and aberrant differentiation toward cartilage and bone, with persistently impaired function (59). The authors showed that adult Scx-lineage tenocytes are not recruited into the tendon defect but transdifferentiate into ectopic cartilage, which in turn leads to the infiltration of extrinsic α-smooth muscle actin (α-SMA)-expressing cells that persist to form a permanent scar (59). This was attributed to the fact that, in adult tendons, although tenocytes appeared to be activated in response to injury, there might be an abnormal tendency toward chondrogenic differentiation, leading to a compromised tenogenic differentiation process and consequently no contribution to the healing of the tendon defect (59). These results were also confirmed by Komura et al., who showed complete tendon healing in neonatal mice, different from adult mice that showed signs of chondrometaplasia at tendon stumps and α-SMA-expressing myofibroblast-induced scar healing (61). Furthermore, it has been demonstrated that chondrometaplasia occurrence at the expense of tenogenic differentiation during tendon healing in adult mice can also be attributed to the downregulation of *MKX* expression, unveiling its potential inhibitory effect on chondrogenic differentiation in tenocytes (59).

Of note, chondrometaplasia is observed in both mice and humans during spontaneous tendon healing, offering valuable insights into the underlying mechanisms and potential therapeutic approaches and making mice an ideal model for studying tendon healing and translating the findings to human patients. Investigating the molecular and cellular processes involved, and evaluating the efficacy of interventions might offer new perspectives to better understand how to promote proper tendon healing in humans. Additionally, mouse models allow for controlled experiments and genetic manipulations that can help unravel the complexities of chondrometaplasia. Overall, understanding the mechanisms underlying tendon injuries and chondrometaplasia is essential for developing effective strategies to prevent, diagnose, and treat these conditions, ultimately serving as a valuable bridge for translating discoveries into clinical applications for the treatment of tendon injuries in humans.

In summary, this study emphasizes the efficacy of the DVC monitoring system in predicting tendon ECM remodeling and subsequent functional recovery in a mouse model. It is crucial to recognize, however, that the current application of the DVC monitoring system is restricted to small animals, specifically mice, and is not suitable for larger animals such as sheep or horses. Unfortunately, in compliance with animal experimentation authorization, the inclusion of female mice, which could provide deeper insights into gender-specific effects on tendon healing, was not feasible in this study. Additionally, it is worth acknowledging that the induced tendon lesion in this study was mechanically generated and, therefore, the obtained results may not fully capture the diverse range of lesions observed in individuals affected by tendinopathies.

## 5 Conclusion

The present study introduces a novel approach to predict mice tendon structure recovery by utilizing high-resolution spatiotemporal data on activity patterns obtained through continuous, non-invasive home-cage monitoring.

The findings indicate a positive correlation between locomotor activity and key quantitative morphometric parameters, shedding light on the processes of cell alignment and ECM remodeling during the early stage of the tendon healing process. Moreover, the study emphasizes the positive impact of unrestricted running on wheels on accelerating the recovery of mice tendon microarchitecture. This effect was particularly pronounced in animals with bilateral tendon injuries, likely a result of the distinct adaptive movement imposed by the bilateral mechanical tissue defects.

The obtained results demonstrate that the 24/7 home-cage monitoring activity was sensitive and reproducible enough to couple the movement patterns with the degree of tendon healing. This non-invasive continuous approach overcomes the preclinical limit of traditional sporadic monitoring methods, offering deeper insights into the complex processes of mice tendon homeostasis recovery.

The robustness of the monitoring set-up offers new perspectives on the complex mechanisms underlying mice tendon healing across various conditions: physiological (healthy), paraphysiological (comparing aged models to young ones and studying animals with different lifestyles or exposed to pharmacological interventions), and pathological (metabolic diseases and transgenic mice).

## Data availability statement

The raw data supporting the conclusions of this article will be made available upon request by the authors, without undue reservation.

## Ethics statement

The animal study was approved by Italian Ministry of Health with the authorization number 183/2021-PR on 12/03/2021. The study was conducted in accordance with the local legislation and institutional requirements.

## Author contributions

MF: Formal analysis, Investigation, Writing – original draft. MEK: Data curation, Formal analysis, Investigation, Writing – original draft. VR: Conceptualization, Funding acquisition, Methodology, Supervision, Validation, Visualization, Writing – review & editing. MRi: Data curation, Formal analysis, Writing – original draft. MRa: Conceptualization, Supervision, Validation, Visualization, Writing – review & editing. OG: Data curation, Investigation, Writing – review & editing. PB: Supervision, Visualization, Writing – review & editing. AM: Data curation, Investigation, Methodology, Supervision, Visualization, Writing – review & editing. FS: Conceptualization, Investigation, Supervision, Writing – review & editing. FB: Supervision, Validation, Writing – review & editing. VM: Supervision, Visualization, Writing – review & editing. LV: Methodology, Writing – review & editing. BB: Conceptualization, Funding acquisition, Supervision, Validation, Visualization, Writing – review & editing.
