# Supplementary material for: Unraveling the link: locomotor activity exerts a dual role in predicting Achilles tendon healing and boosting regeneration in mice
Source: Front Vet Sci. 2023 Dec 21;10:1281040. doi: 10.3389/fvets.2023.1281040 (PMC10764449; doi:10.3389/fvets.2023.1281040)
Supplement: Supplementary file 1 [file Table_1.docx]

**Supplementary Table 1. Inter-assessor reliability assessment of the semi-qualitative histological parameters conducted by analyzing percent agreement (%), Cohen’s kappa scores, and weighted kappa scores.**

| Semi-qualitative parameter | Percent agreement (%) | Cohen’s kappa (κ) score | Interpretation of κ score | Weighted kappa (κ_w_) score |
| --- | --- | --- | --- | --- |
| COL1 fiber alignment | 87.87% | 0.827 | Almost perfect agreement | 0.881 |
| Vascularity | 81.25% | 0.728 | Substantial agreement | 0.846 |
| Chondrometaplasia | 93.94% | 0.786 | Substantial agreement | 0.804 |
| Osteometaplasia | 84.85% | 0.680 | Substantial agreement | 0.716 |
